# A mathematical investigation of polyaneuploid cancer cell memory and cross-resistance in state-structured cancer populations

**DOI:** 10.1038/s41598-023-42368-8

**Published:** 2023-09-12

**Authors:** Anuraag Bukkuri, Kenneth J. Pienta, Robert H. Austin, Emma U. Hammarlund, Sarah R. Amend, Joel S. Brown

**Affiliations:** 1 Cancer Biology and Evolution Program and Department of Integrated Mathematical Oncology, Moffitt Cancer Center, Tampa, USA; 2 The Brady Urological Institute, Johns Hopkins School of Medicine, Baltimore, USA; 3 Department of Physics, Princeton University, Princeton, USA; 4 Tissue Development and Evolution Research Group, Department of Laboratory Medicine, Lund University, Lund, Sweden

**Keywords:** Cancer therapy, Ecological modelling, Evolutionary ecology, Population dynamics, Theoretical ecology, Evolutionary theory

## Abstract

The polyaneuploid cancer cell (PACC) state promotes cancer lethality by contributing to survival in extreme conditions and metastasis. Recent experimental evidence suggests that post-therapy PACC-derived recurrent populations display cross-resistance to classes of therapies with independent mechanisms of action. We hypothesize that this can occur through PACC memory, whereby cancer cells that have undergone a polyaneuploid transition (PAT) reenter the PACC state more quickly or have higher levels of innate resistance. In this paper, we build on our prior mathematical models of the eco-evolutionary dynamics of cells in the 2N+ and PACC states to investigate these two hypotheses. We show that although an increase in innate resistance is more effective at promoting cross-resistance, this trend can also be produced via PACC memory. We also find that resensitization of cells that acquire increased innate resistance through the PAT have a considerable impact on eco-evolutionary dynamics and extinction probabilities. This study, though theoretical in nature, can help inspire future experimentation to tease apart hypotheses surrounding how cross-resistance in structured cancer populations arises.

## Author summary

Therapeutic resistance remains one of the major contributors to treatment failure in cancer patients. Studies show that a poly-aneuploid cancer cell (PACC) state may promote resistance by providing a refuge from therapy and increasing the generation of heritable variation, upon which evolution acts. Furthermore, recent evidence suggests that post-therapy PACC derived recurrent population display high levels of cross-resistance to different classes of therapies.

In this paper, we construct a mathematical model, inspired by life history theory, to examine how this may occur. We show how this cross-resistance may be the result of a PACC memory, wherein cancer cells that have entered the PACC state can re-enter it more quickly upon subsequent insult, or an innate resistance, in which cancer cells that have undergone a poly-aneuploid transition have a higher innate resistance to other therapies. We also show how resensitization dampens the effectiveness of innate resistance to produce cross-resistance. This work will guide future experimental research to elucidate mechanisms of cross-resistance and may also inform drug development studies to target aspects of PACC biology.

## Introduction

Cancer research and clinical oncology have made great strides in the last several decades, from advances in the basic science of cancer biology to revolutions in genomics and personalized medicine to the development of cutting-edge therapies^1–11^. However, despite this progress, therapeutic resistance remains a major contributor to treatment failure in cancer patients^12–19^. Therapeutic resistance arises through a process of Darwinian evolution: Therapy is applied to a population of cancer cells and some of these cells harbor or develop mutations that afford them some level of resistance to the stressor^20^. This resistance provides the cells with a higher fitness (defined here as per capita growth rate)^21^. In a process of creative destruction, cells with higher fitness proliferate at the expense of less fit cells. In this way, resistance evolves and eventually the cancer therapy becomes ineffective^22^.

Experimental evidence indicates that a polyaneuploid cancer cell (PACC) state plays a key role in this process. Cells in the 2N+ state (aneuploid cancer cells with abnormal numbers of chromosomes or chromosomal fragments that display structural rearrangements, amplifications, and deletions^23–25^) can enter the PACC state^24,26–28^ (undergoing what we term a polyaneuploid transition or PAT) by undergoing endocycling^29^, thereby accruing greater than G2 genomic material^24,26,30^. At the cost of being non-proliferative, this state allows cells to persist under stressful conditions^31^ and to increase their capacity to generate heritable variation (evolvability)^32–36^ due to the higher amount of genomic material that precipitates from the polyploidization program^37–40^. In other words, unlike traditional polyploid cells and cell fusions that can divide within the polyploid state^41^, the PACC state is a state in the life history of a cancer cell. Cells cannot divide within this state, but must enter the 2N+ state via depolyploidization to divide. In prior work, we used a modeling framework we developed^42^ to understand how therapy affects the dynamics of the 2N+ and PACC populations (ecology) and resistance strategies (evolution)^43,44^. However, one aspect lacking in this modeling is a careful consideration of how cancer cell populations respond to subsequent therapeutic insults with the same or different classes of therapy. In an ongoing study by Amend and Pienta, prostate cancer cell lines were exposed to docetaxel therapy for 72 hours. After confirming that the surviving population was primarily composed of PACCs, the cells were isolated and allowed to transition to a primarily non-polyploid state. These progeny were then rechallenged with another therapy (cisplatin, radiation, or docetaxel). In each case, the population displayed higher levels of resistance to the second stressor than would be expected in control cancer cell populations that had not been exposed to the first round of therapy (Amend & Pienta, pers comm). However, the mechanisms by which this cross-resistance occurs remain opaque.

In this paper, we build on previously created mathematical models of 2N+ and PACC eco-evolutionary dynamics^43,44^ to investigate two hypotheses for how this cross-resistance occurs. Our first hypothesis is called “PACC memory”. This hypothesis posits that cancer cells that have entered the PACC state retain a “memory” of the PAT, which they can access to rapidly transition to the PACC state upon subsequent insult. In this way, PACC memory affords the cancer cell population a stress-agnostic mechanism of multi-drug resistance. Our second hypothesis is called “innate resistance”. This hypothesis contends that cancer cells that have undergone a PAT have a higher innate resistance to other therapies. We also allow for the possibility that this innate resistance is transient. We explore how key parameters (transition rate to the PACC state, innate resistance levels, and resensitization rates) impact extinction probabilities. We show how PACC memory and innate resistance can both lead to high levels of cross resistance and that resensitization of 2N+ cells has a considerable impact on extinction probabilities. In addition to the basic science value of this study to mathematical modeling and cancer biology, understanding the causes of cross-resistance in cancer cell populations can help effectively guide future drug discovery and therapeutic strategies.

## Models

### ODE model: capturing ecology

To construct our eco-evolutionary model, we begin by building an ODE model that captures key ecological dynamics of cells in the 2N+ state, cells in the PACC state (PACC), and 2N+ cells that have previously undergone a PAT (2N+(R)). This model, shown in Equation 1, builds on prior work^43,44^ by incorporating a 2N+(R) state and allowing for resensitization.

1 $$\begin{aligned} \begin{aligned} \frac{dN}{dt}&= \underbrace{rN\left( \frac{K-N-P-R}{K}\right) }_\text {Logistic Growth}\overbrace{-\gamma N}^\text {Obligate to PACC}{-\sum _i\Bigg (}\underbrace{N\frac{m}{\lambda _N+\beta v_i}}_\text {Drug-Induced Death}\overbrace{+c_{N}N\frac{m}{\lambda _N+bv_i}}^\text {Facultative to PACC}{\Bigg )}\underbrace{+\mu R}_\text {Resensitization from 2N+(R)}\\ \frac{dP}{dt}&= \underbrace{\zeta \gamma (N+R)}_\text {Obligate from 2N+ and 2N+(R)}\overbrace{+\zeta \sum _i\left( c_{N} N\frac{m}{\lambda _N+\beta v_i}+c_{R}(\textbf{v}) R\frac{m}{\lambda _R(\textbf{v})+\beta v_i}\right) }^\text {Facultative from 2N+ and 2N+(R)}\underbrace{-aP}_\text {To 2N+(R)}\overbrace{-bP}^\text {Background Death}\\ \frac{dR}{dt}&= \underbrace{rR\left( \frac{K-N-P-R}{K}\right) }_\text {Logistic Growth}\overbrace{-\gamma R}^\text {Obligate to PACC}{-\sum _i\Bigg (}\underbrace{R\frac{m}{\lambda _R(\textbf{v})+\beta v_i}}_\text {Drug-Induced Death}\overbrace{+c_{R}(\textbf{v})R\frac{m}{\lambda _R(\textbf{v})+\beta v_i}}^\text {Facultative to PACC}{\Bigg )}\\&\underbrace{+2aP}_\text {From PACC}\overbrace{-\mu R}_\text {Resensitization to 2N+} \end{aligned} \end{aligned}$$

In this model, *N*, *P*, and *R* represent the population sizes of cells in the 2N+, PACC, and 2N+(R) states and $\textbf{v}=[v_1,v_2]$ is the vector of drug resistance strategies where $v_i$ represents the resistance strategies of the cells to drug *i*. Note that the resistance levels, $v_i$, are population-level traits that capture the susceptibility of cells in the 2N+ and 2N+(R) states to therapy. Although the trait is impacted by transitions into and from the PACC state, it does not consider the temporary resistance cells obtain in the PACC state as part of the evolving resistance. In other words, $v_i$ isolates the genetic evolution of cells to therapy and avoids confounding this with the plastic transitions to the PACC state. Although most theoretical models only allow for either genetic evolution or plastic state-switching^21,45–47^, our model is able to incorporate both mechanisms of adaptation and evolution to the therapeutic stressor.

We assume that cells in the 2N+ and 2N+(R) state grow in a logistic manner, equally inhibited by cells in the 2N+, PACC, and 2N+(R) states, with intrinsic growth rate *r* until they reach their carrying capacity, *K*. We allow for an obligate transition rate to the PACC state ($\gamma$), an assumption supported by experimental literature that shows a baseline level of cells in the PACC state in non-treated populations^26,48–56^. We incorporate death due to therapy in a Michaelis-Menten fashion for cells in the 2N+ and 2N+(R) states. Under this formulation, death depends on drug dosage (*m*), innate resistance ($\lambda _{N,R}$), the evolving resistance trait ($v_i$), and a scaling term to capture the impact of evolving higher resistance levels ($\beta$)^21,57–59^.

Furthermore, we include a condition-dependent transition ($c_{N,R}$) to the PACC state: The higher the death due to therapy, the higher the transition rate will be. Such facultative transitions are noticed in experimental studies where PACC frequency and number increase upon the initial administration of a variety of therapies, including hypoxia, chemotherapy, and radiation therapy^26,48–51,54,55,60–64^. For each transition into the PACC state, we include a probability of successful PAT ($\zeta$) since unsuccessful PAT due to mitotic catastrophe and subsequent cell death is possible. The 2N+(R) state also receives cells from the PACC state in an obligate manner (*a*) and we allow resensitization ($\mu$) of 2N+(R) cells into 2N+ cells under our temporary innate resistance hypothesis.

**Figure 1:**
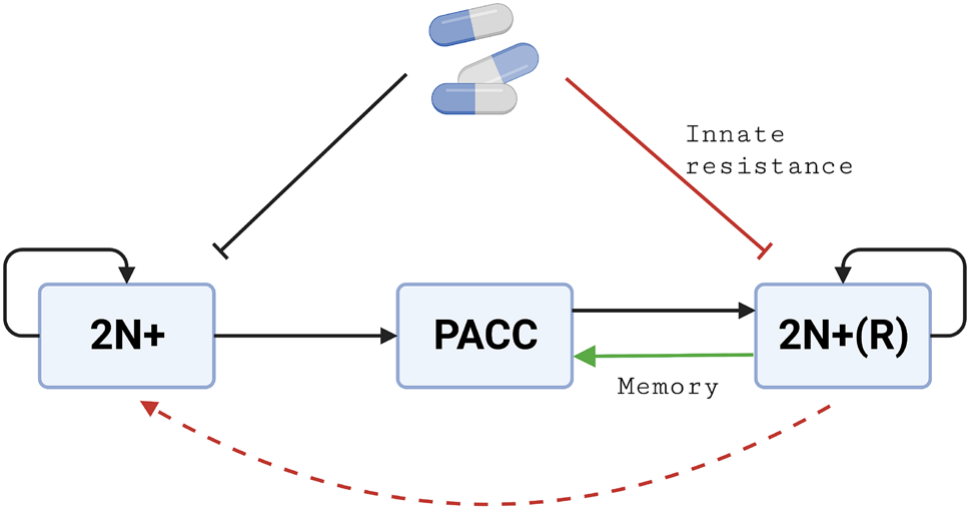
Life Cycle Graph for 2N+, PACC, and 2N+(R) Transitions. Cells in the 2N+ and 2N+(R) states can self-replicate or undergo a PAT into to the PACC state. Cells in the PACC state can depolyploidize into the 2N+(R) state. Under the PACC memory hypothesis, 2N+ cells that have undergone PATs due to therapy can transition into the PACC state more quickly (green arrow). Under the innate resistance hypothesis, these cells instead display higher rates of innate resistance to therapeutic stressors (red inhibitory line). If innate resistance in the 2N+(R) state is transient, cells in the 2N+(R) state can be re-sensitized to the 2N+ state (red dashed arrow). Created with BioRender.com.

Cells in the PACC state are assumed to be non-proliferative and fully resistant to therapy^24,26,54^, so their dynamics are simply given by transitions from the 2N+ and 2N+(R) states, transitions back to the latter, and a background death rate (*b*). We let $\lambda _R(v) = \frac{\lambda _R}{1+exp(-2\sum _iv_i)}$ and $c_R(v) = \frac{c_R}{1+exp(-2\sum _iv_i)}$ increase as a function of drug resistance in a logistic manner. Note that this model does not include a cost of resistance and assumes infinite improvement in which the fitness gradient with respect to drug resistance is always positive. The transitions between cell states in this model can be seen in Figure 1. The interpretations of all parameter values and baseline levels used in our simulations can be found in Table 1.

**Table 1:** Parameter definitions and values used in simulations.

Parameter	Interpretation	Value
*r*	Intrinsic growth rate	0.6 day$^{-1}$
*K*	Carrying capacity	100*$10^7$ cells
$\gamma$	Obligate transition rate	0.02 day$^{-1}$
*m*	Drug Dosage	0.7 day$^{-1}$
$\lambda _N$	Innate resistance: 2N+	1
$\lambda _R$	Innate resistance: 2N+(R)	2
$\beta$	Efficacy of evolving a resistance strategy	1
$c_{N}$	Facultative transition scaling rate: 2N+	0.4 day$^{-1}$
$c_{R}$	Facultative transition scaling rate: 2N+(R)	0.8 day$^{-1}$
*a*	PACC to 2N+(R) transition rate	0.2 day$^{-1}$
*b*	Background PACC death rate	0.01 day$^{-1}$
$\mu$	Resensitization rate	0.2 day$^{-1}$
$\zeta$	Successful polyaneuploid transition rate	0.7 day$^{-1}$
$\phi$	Mutation rate	0.05 division$^{-1}$
$\sigma _1$	Breadth of mutation: 2N+ & 2N+(R) Division	0.01
$\sigma _2$	Breadth of mutation: PACC Depolyploidization	0.05
$v_i$	Evolving trait: resistance to drug *i*	$[0,\infty )$

The parameter values were replicated from our prior work^43,44^ and chosen to be biologically plausible and numerically convenient to show clear differences between the control, PACC memory, and innate resistance hypotheses. The results of sensitivity analyses for key parameters of the model can be seen in the supplemental material in^44^. Broadly, we find that for low growth rates, the cancer cell population cannot divide fast enough to avoid therapy-induced extinction. Conversely, high growth rates allow the cancer cell population to evolve resistance fast enough and undergo evolutionary rescue to avoid extinction. We find that a higher obligate transition rate reduces the probability of extinction by maintaining a higher frequency of cells in the PACC state at baseline. Similarly, higher facultative transitions to the PACC state promote survival under therapy as cells are more quickly able to shift into the PACC state and avoid the effects of therapy. Intuitively, as the efficacy of the resistance strategy is increased, extinction probability decreases since the effects of therapy are more rapidly minimized due to the evolution of resistance. Finally, we discovered that mutation rate displays a “Goldilock’s effect”, wherein low and high mutation rates lead to extinction since cells cannot evolve resistance fast enough or undergo mutational meltdown, respectively. A medium mutational burden balances these aspects, providing enough fuel for genetic evolution while preventing too many failed divisions from occurring. Although results quantitatively vary with different parameter values, we find that the same qualitative trends hold.

### Stochastic implementation: incorporating evolution

To simulate the dynamics of our cancer cell population from our ODE system in Equation 1 and incorporate evolutionary processes, we use a birth-death-switching process introduced in earlier work^44^ that is largely based on the Gillespie algorithm. This approach is similar to Dieckmann’s directed random walks in adaptive dynamics^65–67^ whereby evolution proceeds as a sequence of trait substitutions with each selected mutation conferring a positive invasion fitness in the resident population^68^. It is critical to note that our simulation is not an agent-based model, but rather implements the stochastic aspect at the population level. Thus, homogeneity within states is assumed and cells are not endowed with individual properties.

Our simulation procedure (identical to the one outlined in^44^) for ecological dynamics follows the Gillespie algorithm. Within this framework, we added a mutational component to incorporate evolutionary dynamics. Namely, we initialize the population with 50 cells in the 2N+ state and with $v_1=v_2=0$. For each event, we directly read off the birth, death, and switching rates from Equation 1. This gives us nine different rates: birth in the 2N+ state, birth in the 2N+(R) state, death in 2N+ state, death in the PACC state, death in the 2N+(R) state, switching from 2N+ to PACC, from PACC to 2N+(R), from 2N+(R) to PACC, and from 2N+(R) to 2N+. We then sum these rates to obtain a total event rate. From this, the time to next event is determined by sampling from an exponential probability distribution with a mean of the total event rate. The greater the number of potential events (e.g., due to a higher population size) or the higher chance of events happening (e.g., due to a higher intrinsic growth rate), the shorter the time to next event will be. Based on the contributions of each type of event, an event is chosen and carried out. If the event is death, one cell of the corresponding cell type will be eliminated. If the event is birth, one cell of the corresponding cell type will be added to the population. Events will continue to be executed until the time elapsed for the simulation reaches 900.

During cell division, we allow for a chance of mutation with rate $\phi$. Specifically, we sample the additive value of the mutation from a Gaussian distribution with mean 0 and breadth $\sigma _1$. If the resulting mutant has a lower fitness than those of the residents, it is purged from the population. However, if the mutant has a higher fitness, it remains in the population and, invoking the invasion implies substitution theorem from adaptive dynamics^69,70^, its trait value replaces the resident trait value. Through this process, evolution is introduced into our model. This update is done immediately, implying that fixation of a mutant occurs instantaneously, whereas a mutant is generated once every 20 cell divisions, on average. Under this formulation, the drug resistance trait can only change during times of therapy–it is fixed (i.e. does not drift) during drug holidays. Our simplistic mean-field approximation of the evolutionary process implies that, in the absence of exogenous factors, fitness is non-decreasing. In reality, however, genetic drift may reduce the fitness of populations at steady-state, a consideration we ignore here for simplicity. Furthermore, each cell may be endowed with a different fitness, a consideration that may influence the trajectory of the ecological and evolutionary dynamics of interacting cells in the population. We ignore the effects of such heterogeneity in this model for simplicity.

If the event is switching from the 2N+ or 2N+(R) state to the PACC state, one cell of the former will be replaced by one cell of the latter. Similarly, if the switching event occurs from the 2N+(R) to the 2N+ state, we replace one 2N+(R) cell with one 2N+ cell. If the event is switching from the PACC to 2N+(R) state, we replace one cell in the PACC state with two cells in the 2N+(R) state. During this depolyploidization event, we also allow for the possibility of a mutation to arise. To implement this, we follow the same process as for birth events, but we use $\sigma _2$ instead of $\sigma _1$ for the breadth of the mutation. This larger mutational breadth is presumed to be the result of the higher genomic material characteristic of cells in the PACC state. Thus, there are two pathways by which cells can gain resistance. Cells in the 2N+ and 2N+(R) state can directly mutate upon division and give rise to resistance or they can enter the PACC state and mutate upon depolyploidization. The only difference between these two pathways is that cells in the PACC state do not die from therapy and have the potential to acquire more extreme mutations when they depolyploidize due to the higher mutational breadth.

## Results

With this algorithm for stochastically simulating the eco-evolutionary dynamics of our cancer cell population, we examined questions surrounding the response of these populations to repeated therapeutic insults. We simulated several hypotheses on how cross-resistance may emerge in cancer cell populations. In each case, for 10 sets of 100 trials, we ran the simulation until time 900: no therapy until time 100, drug 1 until time 400, no therapy until time 500, drug 1 or 2 until time 800, and no therapy until time 900. One set of these simulations was plotted to visualize eco-evolutionary dynamics over time and mean and standard deviation extinction probabilities were reported across these sets. We investigated effects of drug dosage and key parameters such as transition to the PACC state, level of innate resistance, and resensitization rate that are central to the formulation of each hypothesis. In each of the following simulations, the top panel captures the population dynamics and the bottom panel captures the resistance strategy dynamics. Red, blue, and green curves represent cells in the 2N+, PACC, and 2N+(R) states respectively. Black and magenta curves depict the resistance strategies to drug 1 and drug 2 respectively. Backgrounds shaded in yellow and pink represent when drug 1 or drug 2 are being administered respectively.

### Control

**Figure 2:**
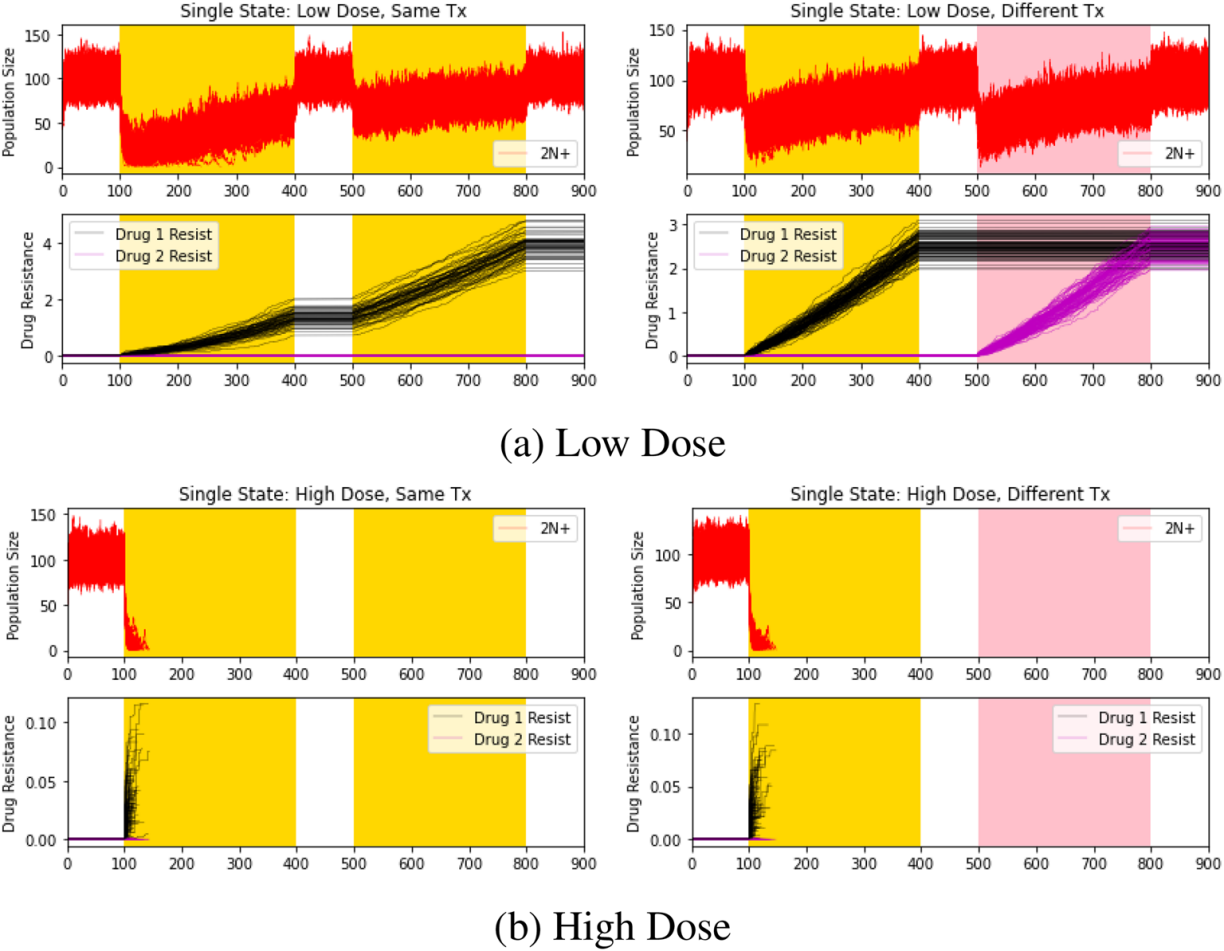
Single state: effects of dosage and therapy type. Red, black, and magenta lines depict 2N+ population dynamics, drug 1 resistance dynamics, and drug 2 resistance dynamics, respectively. Areas shaded in yellow and pink capture periods when drug 1 and drug 2 are administered, respectively. Higher extinction probabilities were observed for higher doses and different therapies.

Before exploring mechanisms of cross resistance, we simulate various control cases. First, we consider the single-state case in which the cancer cell population lacks a PACC state entirely: the population exists in a single 2N+ state. To do this, we removed the PACC and 2N+(R) states and simulated eco-evolutionary dynamics under the same and different classes of drugs with low ($m=0.5$) and high ($m=0.7$) doses (Figure 2).

First, consider the low dose simulation results in Figure 2a. As expected, the cancer cell population was significantly more effective at avoiding extinction when exposed to repeated application of the same drug than when exposed to different drugs (the population went extinct in $40\pm 7.0\%$ and $66.1\pm 5.8\%$ of trials for same and different therapies, respectively: $p<0.0001$). This is because when the same therapy is administered twice, the second application of therapy is less effective as the cancer cell population has already gained resistance from the first therapeutic cycle. Thus, all extinctions come during the first administration of therapy. On the other hand, since drug 1 and drug 2 act independently (i.e., resistance to drug 1 does not affect resistance to drug 2) and are given at the same dose, the second administration of therapy has similar effects as the first one and is equally effective at decimating the population. We see the evolution of resistance to drugs 1 and 2 in the bottom panels, as the resistance strategy gradually increases during times of therapy and remains static during times of no therapy.

Now, consider the high dose case. Regardless of whether the same or different therapies were administered, the cancer cell populations were not able to evolve resistance fast enough to remain extant, going extinct in all sets of trials during the first application of therapy (Fig. 2b). Cancer cell populations existing in a single 2N+ state could not evolve resistance fast enough to avoid extinction in the face of high initial therapy efficacy.

**Figure 3:**
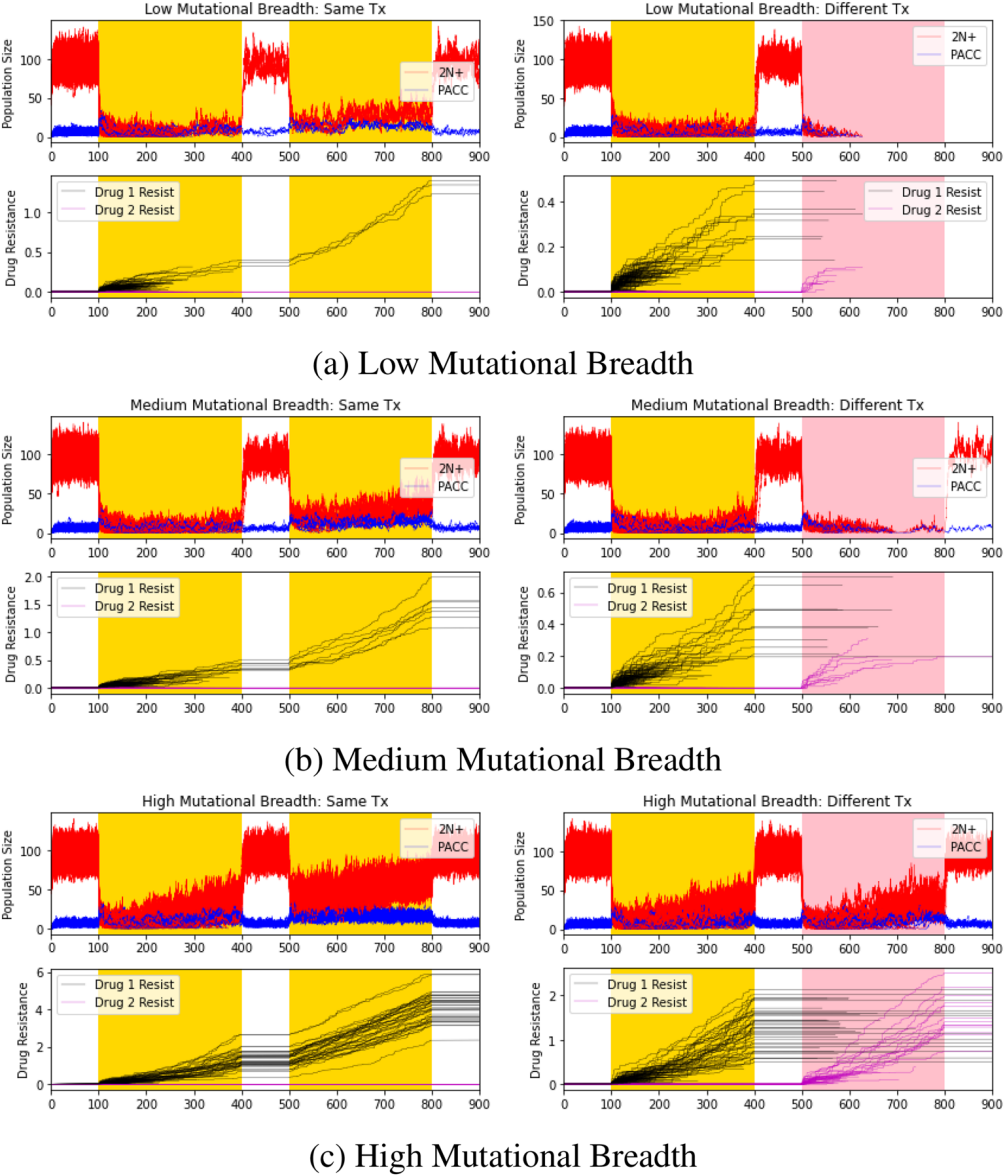
PACC model: effects of mutation breadth and therapy type. Red, blue, black, and magenta lines depict 2N+ population dynamics, PACC population dynamics, drug 1 resistance dynamics, and drug 2 resistance dynamics, respectively. Areas shaded in yellow and pink capture periods when drug 1 and drug 2 are administered, respectively. High mutational breadth promotes evolutionary rescue via rapid evolution. Population had a lower extinction probability when exposed to the same therapy than when exposed to different therapies.

This leads to the question of how the PACC state, which provides a refuge from extreme environmental conditions and may increase heritable variation in the population, can help promote therapeutic resistance? To address this question, we ran another set of simulations, this time allowing for transitions into and from the PACC state. We did not allow for any mechanisms of cross-resistance, i.e., we removed the 2N+(R) state entirely. For the same and different classes of therapies, we examined the impact of low ($\sigma _2=0.005<\sigma _1$), medium ($\sigma _2=0.01=\sigma _1$), and high ($\sigma _2=0.05>\sigma _1$) mutational breadths of the PACC state on eco-evolutionary dynamics (Fig. 3). To see the clearest difference between the single state hypothesis and the following hypotheses, we used a high dose of therapy for the remainder of the simulations.

**Table 2:** Effects of mutation breadths and therapy type on extinction probabilities.

	Same therapy (%)	Different therapies (%)
Low breadth	$95.2\pm 2.4$	$99.8\pm 0.4$
Medium breadth	$91.9 \pm 2.7$	$99.3 \pm 0.7$
High breadth	$63.2\pm 5.7$	$87.3\pm 3.2$

The extinction probabilities in each of these simulations are shown in Table 2. With higher mutational breadth, there are fewer extinction events ($p<0.0001$ for each pairwise comparison). This is because, as can clearly be seen in the evolutionary dynamics, the cell population is able to evolve resistance at a faster rate and avoid extinction. Similar to our single-state case, we note that the population performed better when exposed to the same therapy than different therapies ($p<0.0001$). In accord with experimental studies^26,48–56^, we notice a low level of cells in the PACC state pre-therapy. Upon therapeutic administration, we observe a rapid shift in the population from cells in the 2N+ state to cells in the PACC state^26,48–51,54,55,60–64^. Gradually, the frequency of cells in the 2N+ state increases as resistance evolves. When therapy is removed, cells quickly return to their pre-treatment equilibrium, primarily existing in the 2N+ state.

### PACC memory

**Figure 4:**
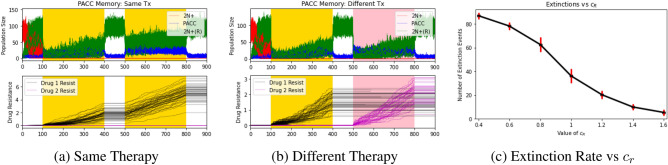
PACC memory: effects of therapy type and transition to PACC state. Red, blue, green, black, and magenta lines depict 2N+ population dynamics, PACC population dynamics, 2N(R)+ population dynamics, drug 1 resistance dynamics, and drug 2 resistance dynamics, respectively. Areas shaded in yellow and pink capture periods when drug 1 and drug 2 are administered, respectively. A rapid retransition into the PACC state can promote cross-resistance in cancer cell populations. The faster this retransition occurs, the lower the extinction probability is.

Next, we simulated the PACC memory hypothesis. We assumed that 2N+ cells that have undergone PATs due to therapy have a “PACC memory” that allows them to retransition to the PACC state more rapidly upon subsequent therapeutic insults. We hypothesized that this rapid transition into the PACC state would allow the cancer cell population to effectively avoid the extreme stressor of therapy and evolve resistance with the help of the PACC state. To simulate this, we let $\lambda _R = \lambda _N$, $c_R(v) = \frac{c_R}{1+exp(-2\sum _iv_i)}$, and $\mu =0$. In this way, the rate of transition to the PACC state is an increasing function of the resistance strategies–the more resistant cells are, the more PATs they have likely undergone, and the faster they are able to switch into the PACC state. In addition to simulations under the same and different therapies, we plotted extinction probabilities over a range of values of $c_R$ in Fig. 4.

Under the PACC memory hypothesis, the cancer cell population went extinct in $53.3 \pm 6.6\%$ and $62.6 \pm 6.2\%$ of trials when exposed to two rounds of the same therapy or two different therapies, respectively ($p<0.0001$). To explore the impact of a more rapid transition to the PACC state more closely, we ran several sets of simulations for values of $c_R$ that range from 0.4 (the baseline $c_N$ rate) to 1.6. We note that nearly all populations remained extant when $c_R$ exceeds 3 times the baseline value. Thus, it is clear that a more rapid transition to the PACC state is a plausible explanation for the higher rates of cross-resistance observed in cancer cell populations.

### Innate resistance

**Figure 5:**
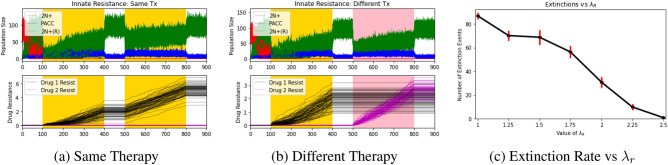
Innate Resistance: Effects of Therapy Type and Level of Innate Resistance. Red, blue, green, black, and magenta lines depict 2N+ population dynamics, PACC population dynamics, 2N(R)+ population dynamics, drug 1 resistance dynamics, and drug 2 resistance dynamics, respectively. Areas shaded in yellow and pink capture periods when drug 1 and drug 2 are administered, respectively. A higher innate resistance in cells that have undergone PATs can promote cross-resistance. The higher this innate resistance is, the lower the extinction probability. Due to the drug-agnostic nature of the innate resistance mechanism, extinction probabilities for populations exposed to the same or different therapies are similar.

To simulate the innate resistance hypothesis, we assumed that 2N+ cells that have undergone PATs due to therapy have a higher innate resistance to future therapies. We hypothesized that this increased innate resistance would provide a therapy-agnostic mechanism of cross-resistance for the cancer cell population. To simulate this, we let $\lambda _R(v) = \frac{\lambda _R}{1+exp(-2\sum _iv_i)}$, $c_R=c_N$, and $\mu =0$. Similar to the PACC memory case, innate resistance was set to be an increasing function of resistance strategies. In addition to simulations under the same and different therapies, we plotted extinction probabilities over a range of values of $\lambda _R$ in Fig. 5.

Under the innate resistance hypothesis, the population went extinct in $28.7\pm 3.0\%$ and $29.2\pm 3.7\%$ of trials when exposed to two rounds of the same therapy or two different therapies, respectively ($p=0.0009$). This minor difference in extinction probabilities between the same and different therapy trials is because the innate resistance mechanism functionally increases the level of resistance in the population in a drug-agnostic way. Thus, although the population still performs better under the same therapy, the difference is less pronounced.

We examined this more finely by running several sets of simulations for values of $\lambda _R$ between 1 (baseline $\lambda _N$ rate) and 2.5. We observed that almost none of the trials resulted in extinction of the population when $\lambda _R$ surpasses 2.25 times the baseline value. Therefore, the innate resistance mechanism can also explain the elevated levels of cross-resistance observed experimentally. It’s worth noting that a less extreme difference between cells in the 2N+ and 2N+(R) states is required for dramatic changes in extinction rate under the innate resistance hypothesis compared to the PACC memory hypothesis.

**Figure 6:**
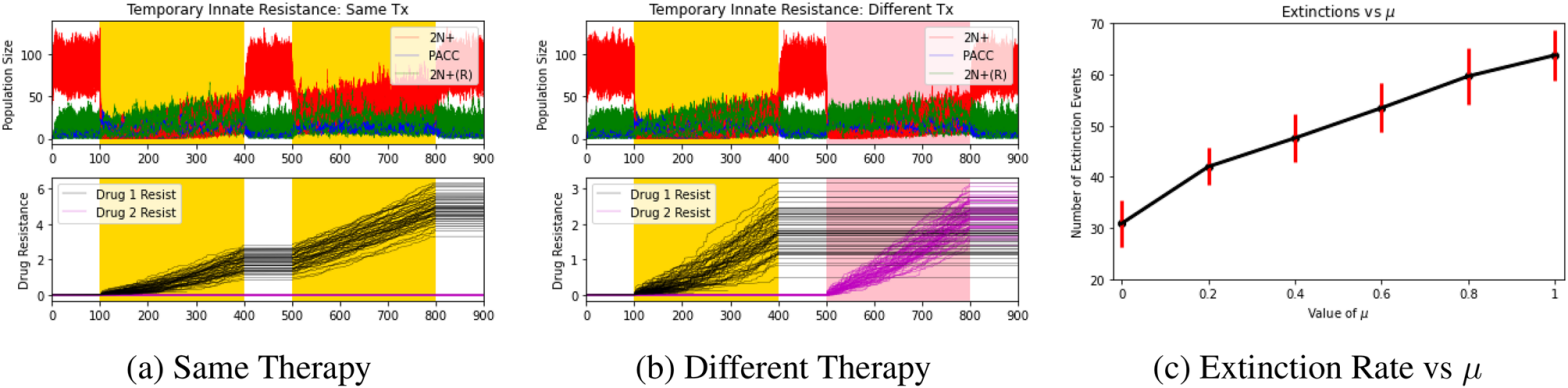
Temporary innate resistance: effects of therapy type and resensitization rate. Red, blue, green, black, and magenta lines depict 2N+ population dynamics, PACC population dynamics, 2N(R)+ population dynamics, drug 1 resistance dynamics, and drug 2 resistance dynamics, respectively. Areas shaded in yellow and pink capture periods when drug 1 and drug 2 are administered, respectively. Resensitization buffers the efficacy of the innate resistance mechanism of cross-resistance.

We next asked the question: what happens if innate resistance is transient? As the temporary innate resistance hypothesis, we allowed for a resensitization rate from cells in the 2N+(R) state to the 2N+ state. To simulate this, we let $\lambda _R(v) = \frac{\lambda _R}{1+exp(-2\sum _iv_i)}$, $c_R=c_N$, and $\mu =0.2$. We hypothesized that accounting for resensitization would increase extinction probabilities as it would buffer the effects of the higher innate resistance of the 2N+(R) state. We again simulated eco-evolutionary dynamics under the same and different therapies and explored the effect of the resensitization parameter on extinction probabilities. The results of these simulations can be seen in Fig. 6.

Under the temporary innate resistance hypothesis, the population went extinct in $39.1\pm 5.7\%$ and $40.1\pm 4.7\%$ of trials when exposed to two rounds of the same therapy or two different therapies, respectively ($p<0.0001$). As expected, resensitization buffered the efficacy of the innate resistance hypothesis to promote cross-resistance. We looked more closely at this by plotting the number of extinction events out of the 100 trials over a range of resensitization rates in Fig. 6c. As we can see, these results confirmed our suspicion that resensitization buffers the effects of an increase in innate resistance: the resulting extinction probabilities, though all lower than the baseline control rates, are higher than those observed under the innate resistance hypothesis.

## Discussion

A major barrier to improved outcomes in cancer treatment is the emergence of resistance. Traditionally, resistance in cancer models has been captured through a single state^20–22,54,71,72^. However, recent theoretical^43,44^ and empirical^24,26–28,54^ studies have demonstrated that the PACC state may play a critical role in the evolution of resistance by allowing cancer cells to persist under extreme stressors and to increase their evolvability. In this paper, we build on our previous theoretical work on modeling the eco-evolutionary dynamics of cancer cell populations with 2N+ and PACC states under therapy by considering how such populations respond to repeated therapeutic insults.

Inspired by preliminary observations by Amend & Pienta, we created a mathematical model to investigate various explanations for how cross-resistance in cancer cell populations may arise. In particular, we considered two major hypotheses: 1) PACC memory, which contends that 2N+ cells that have undergone PATs can reenter the PACC state more rapidly upon subsequent therapeutic insult, and 2) innate resistance, which holds that 2N+ cells that have undergone PATs directly have an increased, stress-agnostic innate resistance. We also considered the possibility for this innate resistance to be temporary by allowing resensitization of cancer cells that have undergone a PAT. We found that although the innate resistance hypothesis was most effective at generating cross-resistance, the PACC memory hypothesis is also a plausible pathway to cross-resistance. We also found that resensitization reduces the effectiveness of innate resistance to produce cross-resistance and aid in the survival of the population. Due to the theoretical nature of our qualitative modeling study, the results are still valid, even if cross-resistance occurs only in limited scenarios, and may be useful to demonstrate general aspects of cross resistance in cancer and medicine.

Overall, this work stresses the importance of considering state-structure in cancer therapeutic resistance to account for both genetic evolution and plastic adaptation. We have shown how the PACC state buffers cancer cells from the effects of therapy and buys them time to evolve resistance. This greatly influences the eco-evolutionary dynamics of cancer cell populations under therapy and must be taken into consideration when designing therapeutic protocols. Namely, as discussed in our earlier work^43^, life history enlightened therapies in which drugs that prevent transition into the PACC state are combined with traditional chemotherapy may be effective at preventing resistance and promoting cancer eradication^73^. We have also demonstrated the plausibility that cancer cells that have undergone PAT may have a memory of the PACC state, allowing them to reenter the PACC state more rapidly when exposed to subsequent stressors. Alternatively, we have shown that cells that have undergone PAT may be imbued with a stress-agnostic mechanism of innate resistance. These findings have important implications for drug scheduling as regimens that administer drugs in quick succession be less effective than those with greater space between treatments. This work has inspired ongoing experimental studies to help tease apart these hypotheses by comparing the rates at which cells in the 2N+ and 2N+(R) states enter the PACC state upon administration of therapy.

## Data Availability

The datasets generated and/or analysed during the current study are available in Anuraag Bukkuri’s GitHub repository, at https://github.com/abukkuri/PACC-Memory.
